# Inhibitory effects of cold atmospheric plasma-activated solution on cutaneous squamous cell carcinoma

**DOI:** 10.3389/fphar.2026.1921696

**Published:** 2026-09-11

**Authors:** Jingfei Wang, Zhengyi Zhang, Baochen Cheng, Xinyi Zhao, Tingyi Yin, Tianji Ni, Wenqian Du, Xinyi Liu, Shuo Feng, Ke He, Jing Zhang, Yan Sun, Dehui Xu, Yan Zheng

**Affiliations:** 1 Department of Dermatology, The First Affiliated Hospital of Xi’an Jiaotong University, Xi’an, China; 2 State Key Laboratory of Electrical Insulation and Power Equipment, Centre for Plasma Biomedicine, Xi’an Jiaotong University, Xi’an, China; 3 School of Life Science and Technology, Xi’an Jiaotong University, Xi’an, China; 4 Department of Dermatology, Tangdu Hospital, Fourth Military Medical University, Xi’an, China

**Keywords:** cutaneous squamous cell carcinoma, cold atmospheric plasma, plasma-activated solution, oxidative stress, apoptosis

## Abstract

Cutaneous squamous cell carcinoma (cSCC) is a common malignant skin neoplasm for which current therapeutic options for advanced and metastatic cases remain limited and are associated with considerable toxicity. Plasma-activated solution (PAS)—prepared by exposing media such as culture medium, water, or phosphate-buffered saline (PBS) to cold atmospheric plasma (CAP)—has emerged as a novel antitumor strategy. In recent years, PAS has garnered increasing attention owing to its advantages over direct CAP application, including ease of storage and clinical administration. In the present study, we investigated the therapeutic potential of PAS in cSCC. In this study, we established an *in vitro* skin cancer model treated with PAS, as well as an *in vivo* xenograft tumor model. *In vivo* experiments demonstrated that PAS significantly suppressed xenograft tumor growth, as evidenced by reductions in both tumor volume and weight, along with a decreased number of Ki-67-positive proliferating cells, without inducing body weight loss or organ damage in treated mice. These antitumor effects were associated with upregulation of the pro-apoptotic protein Bax, downregulation of the anti-apoptotic protein Bcl-2, and suppression of Matrix metalloproteinase-9 (MMP9) expression. *In vitro* assays revealed that PAS elevated intracellular reactive oxygen species (ROS) levels and induced mitochondrial dysfunction, while modulating the expression of apoptosis-related proteins (Bax/Bcl-2) and the migration-associated molecule MMP9. These effects were consistently validated in two squamous cell carcinoma cell lines, HSC-1 and SCL-1. The activation of the NF-κB signaling pathway and subsequent upregulation of pro-inflammatory cytokine expression were also detected in PAS-treated cells. PAS treatment led to tumor cell apoptosis in a time-dependent manner, rather than inducing significant cell cycle arrest. Collectively, PAS exhibits potent antitumor activity without appreciable systemic toxicity. Our findings provide a promising therapeutic approach for cSCC and suggest PAS as a potential strategy for the treatment of other solid tumors.

## 1 Introduction

Among the vast spectrum of malignant neoplasms, cutaneous squamous cell carcinoma (cSCC)—a malignancy originating from squamous epithelial cells—has consistently occupied a position of considerable clinical significance (Liu et al., 2024). As the largest organ of the human body, the skin represents the most common anatomical site for SCC development. Conventional therapeutic modalities for cSCC primarily comprise surgical excision, radiotherapy, and chemotherapy. Surgical resection is indicated for early-stage resectable tumors, enabling direct removal of the lesion (Wang D. M. et al., 2025; Queirolo et al., 2024). Radiotherapy employs ionizing radiation to ablate cancer cells and is frequently utilized for local disease control (Porceddu et al., 2020; Ladwa et al., 2025). Chemotherapy inhibits tumor cell proliferation through cytotoxic agents and is predominantly applied in locally advanced or metastatic cases (Khaddour et al., 2026); however, it is associated with considerable systemic toxicity. In recent years, the incidence of SCC has continued to rise, whereas the therapeutic efficacy of existing treatments remains limited, underscoring the growing imperative for innovative therapeutic approaches with favorable side-effect profiles (Jiang et al., 2026; Spadafora et al., 2025; Thompson et al., 2016; Stratigos et al., 2023).

In recent years, cold atmospheric plasma (CAP) has emerged as a promising physical therapeutic modality in oncology, owing to its non-invasive nature, selective cytotoxicity toward tumor cells, and capacity to induce oxidative stress. As the fourth state of matter, CAP has gained extensive application in the biomedical field (Dai et al., 2025; Almeida-Ferreira et al., 2024; Zhang et al., 2024) and has demonstrated broad antitumor efficacy across multiple cancer types, including neuroblastoma, breast cancer, and cervical cancer (Milesh et al., 2025; Vijayarangan et al., 2020; Wang G. et al., 2025; Qi et al., 2025). In the context of dermatological therapy, CAP has also yielded favorable outcomes in melanoma, atopic dermatitis, and vitiligo (Chen et al., 2024; Yin et al., 2024; Zhai et al., 2021; Zhai et al., 2022; Wu et al., 2024; Rebl et al., 2022).

It is widely accepted that reactive oxygen and nitrogen species (RONS) constitute the principal mediators responsible for plasma-induced biological effects (Liu et al., 2024; Sun et al., 2024; Dai et al., 2021; Friedman, 2020). Plasma-activated solution (PAS)—generated by exposing liquids such as culture medium, water, or phosphate-buffered saline (PBS) to CAP—has emerged as a promising anticancer tool (Qi et al., 2025). CAP generates high concentrations of reactive oxygen/nitrogen species (ROS/RNS) along with electromagnetic fields, which can selectively disrupt mitochondrial function, induce DNA damage, and trigger mitotic catastrophe in tumor cells (Tabassum et al., 2024; Fang et al., 2025; Ya et al., 2020). Accordingly, exposing tissues and cells exclusively to the RONS contained within PAS may represent a safer alternative to direct CAP application (Qi et al., 2025; Yin et al., 2024; Ma et al., 2025).

In the present study, we employed the human cutaneous SCC cell lines HSC-1 and SCL-1 as experimental models to demonstrate that PAS treatment promotes apoptosis, stimulates intracellular ROS generation, and consequently induces mitochondrial damage, thereby suppressing tumor cell migration and invasion. A subcutaneous xenograft tumor model in nude mice was established to investigate the effects of PAS on skin cancer progression. Our findings revealed that PAS induces apoptosis and inhibits migration of cutaneous SCC cells *in vivo*, alongside a comprehensive evaluation of its systemic safety. Collectively, this study demonstrates the therapeutic potential of PAS for cutaneous SCC. Our results provide novel insights into the antitumor potential of PAS in SCC treatment and offer a theoretical basis for the development of precision-based adjuvant therapeutic strategies for cSCC.

## 2 Materials and methods

### 2.1 CAP irradiation apparatus and preparation of PAS

High-purity helium gas (99.999%) was used as the working gas. A mass flow controller (Model 5850 E, Brooks) regulated the gas flow rate at 4 standard liters per minute (SLM). Liquids were treated with a CAP jet at room temperature. The distance between the CAP irradiation apparatus and the liquid surface was maintained at 1.5 cm. In vitro experiments, fresh complete medium supplemented with 10% fetal bovine serum was used as the plasma-activated liquid. For a single treatment, 3 mL of the liquid was placed in a standard 12-well cell culture plate, where the bottom area of each well was 3.6 cm^2^, the liquid depth was 0.79 cm, and the initial temperature of the liquid was 25 °C. The prepared plasma-activated solution (PAS) was used immediately without dilution or storage to replace the original cell culture medium in the wells for a 24-h intervention. The applied volume of PAS in vitro experiments was set as 2 mL per well for 6-well plates, 1 mL per well for 12-well plates, and 0.5 mL per well for 24-well plates. In all in vitro cell experiments using a single treatment condition, PAS was applied to cells for 5 minutes, and then the cells were cultured and functionally tested as described. In vivo experiments, the activated liquid was replaced with sterile PBS buffer with a treatment duration of 5 min, while all other CAP treatment parameters were kept completely consistent with those in the *in vitro* experiments. The freshly prepared PAS was also directly used for intratumoral injection immediately after preparation, without subsequent dilution or storage.

### 2.2 Optical emission spectroscopy (OES)

The gaseous reactive particles generated during discharge are measured by emission spectroscopy. This method uses an optical emission spectrometer (OES, Andor, SR-750i). The fiber probe connected to the spectrometer is placed directly above the vertical line at the discharge outlet for detection. Then, the spectrum is separated by a diffraction grating, and the corresponding emission spectrum can be obtained through the photoelectric conversion of the detector. The wavelength range for measurement is set from 300 to 800 nm, the exposure time is 0.1 s, the gain is set to 2000, and the grating selection is 1,200 grooves/mm.

### 2.3 Quantification of reactive species in activated medium

CAP treatment generates a series of long-lived reactive species, including H_2_O_2_ and NO_2_^−^. H_2_O_2_ was quantified using a hydrogen peroxide assay kit (Beyotime, S0038). Briefly, 50 μL of activated medium treated for varying durations was added to a 96-well plate, followed by 100 μL of the hydrogen peroxide detection reagent per well. After incubation at room temperature for 30 min, absorbance was measured at 560 nm using a microplate reader. NO_2_^−^ was quantified using a nitric oxide assay kit (Beyotime, S0021 S). Briefly, 50 μL of activated medium treated for varying durations was added to a 96-well plate, followed by 50 μL each of Griess Reagent I and Griess Reagent II per well. After incubation at room temperature in the dark for 5 min, absorbance was measured at 540 nm using a microplate reader.

### 2.4 Xenograft experiment

Five-week-old female BALB/c nude mice (purchased from Shanyao Medical Biotechnology Co., Ltd.) were housed in a SPF facility under standard conditions (12 h light/12 h dark cycle, 22 °C ± 2 °C, 50% ± 10% humidity) with free access to sterile food and water. All animal procedures were approved by the Institutional Review Board of Xi’an Jiaotong University (Approval No. XJTUAE 2026-1977). Mice were randomly allocated into 4 groups (n = 5 per group) after 3 days of adaptation, then subcutaneously injected with 200 μL of tumor cell suspension (2.5 × 10^7^ cells/mL). When average tumor volume reached 100 mm^3^, treatment began. All intratumoral injections were completed within 10 s per mouse using a soft fixator, without anesthesia. No pain response or abnormal activity was observed. The plasma-activated solution (PAS) was freshly prepared with 5 min plasma activation. PAS group received daily intratumoral injection of 50 μL fresh PAS for 7 days; vehicle control received 50 μL untreated PBS on the same schedule. Tumor volume was calculated as (Length × Width^2^)/2. All measurements and histological evaluations were independently conducted by a blinded researcher, and the average value of the two measurements was taken as the final data. Humane endpoints were strictly followed: euthanasia when tumor diameter exceeded 15 mm, body weight loss exceeded 20%, or severe ulceration/infection/activity decline occurred. No mice reached the pre-defined humane endpoint ahead of schedule during this experiment.

### 2.5 H&E staining

Following fixation and paraffin embedding, tissue sections were cut at a thickness of 5 μm. Hematoxylin and eosin (H&E) staining was performed according to standard protocols. Sections were scanned using a KF-PRO-005 digital pathology scanner for image acquisition and analysis.

### 2.6 Immunohistochemistry (IHC) analysis

Paraffin-embedded tumor tissues were sectioned at 5 μm and baked in a 65 °C oven for 30 min. Sections were subsequently deparaffinized and rehydrated. Antigen retrieval was performed at high temperature in citrate buffer (pH 6.0). After washing with PBS, endogenous peroxidase activity was quenched with 3% H_2_O_2_ at room temperature. Sections were then washed with PBS and blocked with 10% goat serum for 20 min. IHC staining was performed by overnight incubation at 4 °C with an anti-Ki-67 antibody (1:800). Sections were incubated with a secondary antibody and subsequently stained with a 3,3^′^-diaminobenzidine (DAB) kit at room temperature, followed by counterstaining with hematoxylin. Sections were scanned using a KF-PRO-005 digital pathology scanner for image acquisition and analysis.

### 2.7 Cell culture

The cSCC cell lines HSC-1, and SCL-1 were purchased from Xi’an Youbios Biotechnology Co., Ltd (Xi’an, China) (Du et al., 2026). The cSCC cells were cultured in DMEM and HaCaT cells (ATCC) were cultured in RPMI 1640. Cells were cultured in medium supplemented with 10% fetal bovine serum (FBS) and 1% penicillin–streptomycin at 37 °C in a humidified atmosphere containing 5% CO_2_. We confirmed that all cells are free from *mycoplasma* contamination.

### 2.8 CCK-8 assay for cell viability test

Cell viability was assessed using the Cell Counting Kit-8 (CCK-8) assay (Liji Biotechnology, AC11L054). Measure the cell viability according to the manufacturer’s instructions. Cells were seeded in 96-well plates at a density of 4,000 cells per well and then incubated with PAS treated for varying durations (0, 0.5, 1, 2, 3, 4, or 5 min) for 24 h. After the 24 h continuous culture following PAS treatment, discard the original culture medium in each well, add the mixed system of 10 μL CCK-8 solution and 90 μL fresh complete medium to each well, avoid generating bubbles during the adding process, then place the 96-well plates in a 37 °C, 5% CO_2_ saturated humidity incubator for light-shielded incubation for 1 h, and finally take it out to measure the absorbance at the set detection wavelength with the preheated microplate reader.

### 2.9 Apoptosis detection

HSC-1 and SCL-1 cells were seeded in 12-well plates at a density of 1 × 10^5^ cells per well and cultured for 24 h. Apoptosis was detected using an Annexin V-FITC Apoptosis Detection Kit (Liji Biotechnology, AC12L033). After 24 h of treatment, cells were collected and washed twice with pre-chilled PBS. FITC-conjugated Annexin V and propidium iodide (PI) were added to the cells, followed by incubation at room temperature in the dark for 15 min. Flow cytometric analysis was performed within 1 h.

### 2.10 Cell ROS detection

HSC-1 and SCL-1 cells were seeded in 12-well plates at a density of 1 × 10^5^ cells per well and incubated with PAS. After stimulation, cells were harvested and incubated with DCFH-DA (10 μM; Beyotime, S0033 S) at 37 °C in the dark for 30 min. Cells were then washed twice and resuspended in 200 μL of phosphate-buffered saline (PBS) for flow cytometric analysis (Accuri C6, BD Biosciences) with an excitation wavelength of 490 nm and an emission wavelength of 525 nm. Untreated cells served as controls.

### 2.11 Cell cycle analysis

HSC-1 and SCL-1 cells were seeded in 6-well plates at a density of 2 × 10^5^ cells per well and incubated for 24 h. After stimulation, cells were harvested and fixed overnight with 70% pre-chilled ethanol at 4 °C. RNase A was added to the cell pellets, followed by incubation at 37 °C for 30 min. PI was then added, and cells were incubated at 4 °C in the dark for 30 min. Flow cytometric analysis was subsequently performed (Accuri C6, BD Biosciences). Untreated cells served as controls.

### 2.12 Colony formation assay

HSC-1 and SCL-1 cells were seeded in 6-well plates at a density of 500 cells per well. After attachment, cells were incubated with PAS until macroscopic colonies were visible on the plate bottom. Fresh PAS was replaced every 3 days during this period. The medium was aspirated, and cells were gently washed twice with PBS. Cells were fixed with 4% paraformaldehyde for 20 min, washed with PBS, and stained with 0.1% crystal violet solution (2 mL per well) for 30 min. After PBS washing, images were captured for colony counting, and quantification was performed using ImageJ software.

### 2.13 Scratch assay

HSC-1 and SCL-1 cells were seeded in 6-well plates at a density of 5× 10^5^ cells per well. Upon reaching complete confluence, a linear scratch was created perpendicular to the plate surface using a pipette tip. After PBS washing, cells were cultured in medium containing 5% FBS with PAS.

### 2.14 Protein extraction and western blotting

Cells were lysed in RIPA buffer (P0013C, Beyotime) supplemented with protease inhibitors, followed by ultrasonic disruption, protein quantification, and denaturation. Western blotting analysis was performed as follows: proteins were separated by SDS-PAGE, transferred onto PVDF membranes, blocked with 5% non-fat milk at room temperature for 1 h, and sequentially incubated with primary and secondary antibodies. Chemiluminescent signals were captured using the ChemiDoc imaging system (Bio-Rad) and quantified by densitometric analysis with ImageJ software. The primary antibodies used include: BAX, Bcl-2, MMP9, p-P65, P65, β-actin, and α-tubulin.

### 2.15 RNA isolation and quantitative real-time PCR

Total RNA was extracted from cells using Trizol reagent (Takara, 9,109) according to the manufacturer’s instructions. Reverse transcription was performed to synthesize cDNA from the extracted RNA, which was subsequently used as the template for qPCR. RT-qPCR primers were designed and synthesized by Sangon Biotech (Shanghai, China). qPCR amplification was carried out using PerfectStart Green qPCR SuperMix (Transgene, AQ601) on a Real-Time PCR Detection System (Bio-Rad,CFX-96). Relative gene expression was calculated using the 2^−ΔΔCt^ method, with endogenous controls for normalization. The primer sequences are compiled in Supplementary Table 1, β-actin was employed as a normalization control.

### 2.16 Mitochondrial fluorescence staining

Following PAS treatment, HSC-1 and SCL-1 cells were washed gently three times with pre-warmed PBS. Cells were then incubated with serum-free medium containing MitoTracker Red CMXRos (Beyotime, C1035) and Hoechst 33,342 (Beyotime, C1028) at 37 °C in the dark for 30 min. After incubation, cells were washed three times with pre-warmed PBS to remove unbound dye. Fluorescence images were acquired using a fluorescence microscope.

### 2.17 JC-1 assay for determining mitochondrial membrane potential (MMP)

Mitochondrial membrane potential (MMP) in viable HSC-1 and SCL-1 cells was determined using the lipophilic cationic probe 5,5′,6,6′-tetrachloro-1,1′,3,3′-tetraethylbenzimidazolylcarbocyanine iodide (JC-1, Beyotime, C2006). Post-treatment, cells were gently rinsed with 1× phosphate-buffered saline (PBS), and the JC-1 staining signals were subsequently visualized and assessed under a fluorescence microscope.

### 2.18 Statistical analysis

Data are presented as the mean ± standard error of the mean (SEM). Intergroup differences were analyzed using Student’s t-test for comparisons between two groups, while one-way ANOVA was employed when comparing three or more groups. Statistical significance was defined as *P < 0.05, **P < 0.01, ***P < 0.001, and ****P < 0.0001. Statistical analyses were performed using GraphPad Prism and Origin software.

## 3 Results

### 3.1 Characterization of the CAP irradiation apparatus and PAS

**FIGURE 1:**
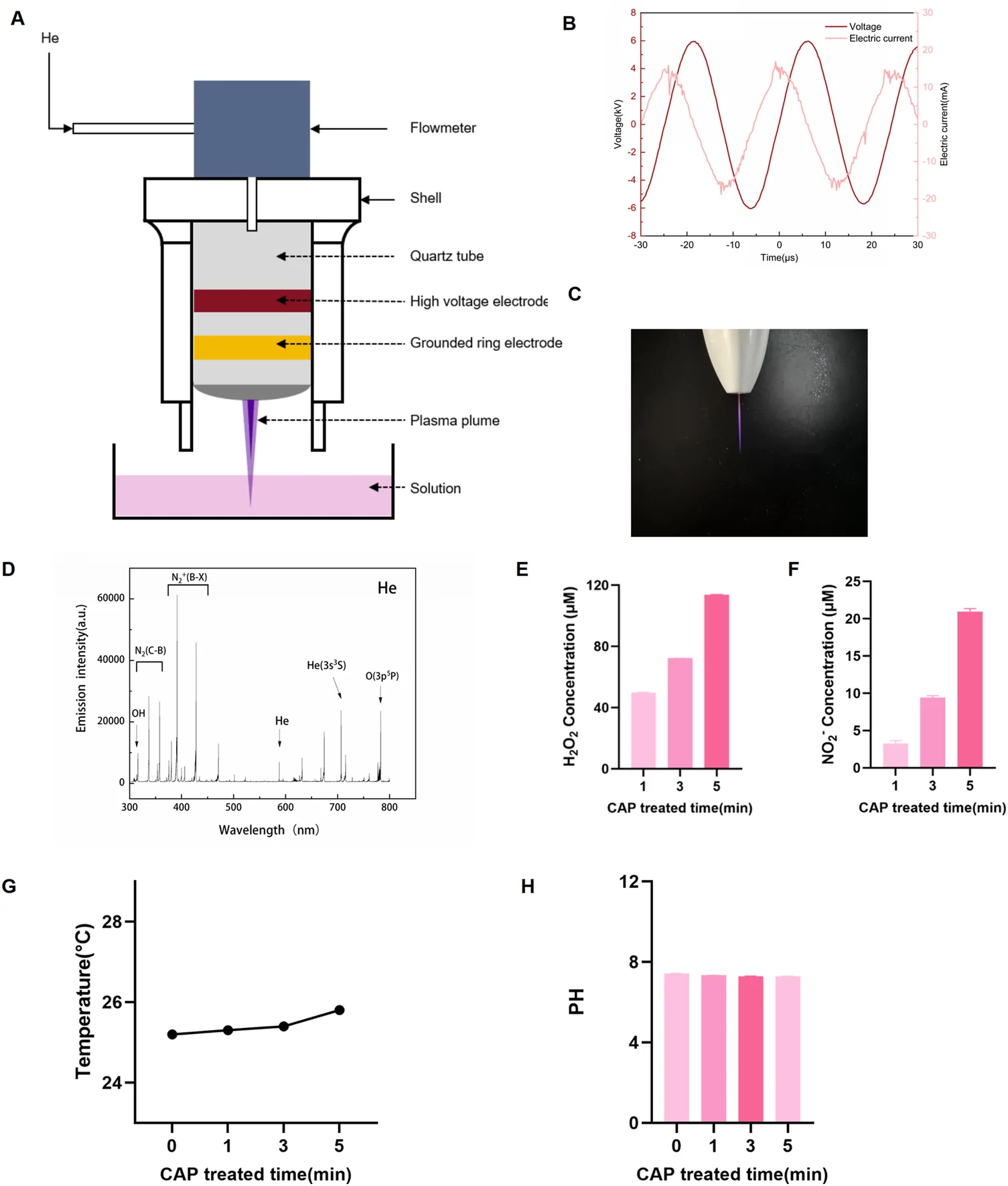
The generation of the CAP irradiation apparatus and the physicochemical properties of PAS. **(A)** Schematic diagram of the CAP irradiation apparatus; **(B)** Voltage and current waveform diagram; **(C)** Photograph of the plasma plume; **(D)** Optical emission spectroscopy (OES) result; **(E,F)** Changes in the concentration of active particles in PAS over the course of CAP treatment; **(G)** The change in temperature of PAS relative to the processing time of CAP; **(H)** The change in pH value of PAS relative to the processing time of CAP.

The portable plasma generation device used in this study is illustrated in Figure 1A. The lower end of the quartz tube features a double-ring discharge configuration. The applied voltage is explicitly defined as the peak-to-peak value of the measured sinusoidal waveform across the two discharge electrodes. During the discharge process, the voltage signal is collected via a high-voltage probe (Tektronix, P6015 A), the current signal is collected via a current probe (Pearson, 2,878), and both signals are recorded by a digital oscilloscope (Tektronix, DPO3052). The maximum voltage is 6 kV, the maximum current is 15 mA, the power supply frequency is 33 kHz, and the average output power is 45 W (Figure 1B).

CAP irradiation apparatus images captured by camera revealed a uniform, slender plasma jet extending approximately 2.5 cm in length (Figure 1C). Optical emission spectroscopy (OES) was employed to identify the atomic and molecular species generated within the CAP jet. The dominant species detected included OH at 309 nm, N_2_ (N_2_ (C–B)), N_2_^+^ (N_2_^+^ (B–Χ)), He, and O (3p^5^P) (Figure 1D). With increasing CAP treatment duration, the concentrations of the long-lived reactive species H_2_O_2_ and NO_2_^−^increased in a time-dependent manner. (Figures 1E,F). Notably, even after the longest CAP treatment, the changes in liquid pH and temperature remain extremely limited: the liquid temperature measured immediately after CAP treatment stabilizes at 25.5 °C ± 0.5 °C (Figure 1G), while the pH value only slightly decreases from 7.4 to 7.2 (Figure 1H), which is close to the physiological conditions and will not introduce confounding factors caused by drastic pH or temperature fluctuations during the treatment.

### 3.2 PAS suppressed xenograft tumor growth *in vivo*

**FIGURE 2:**
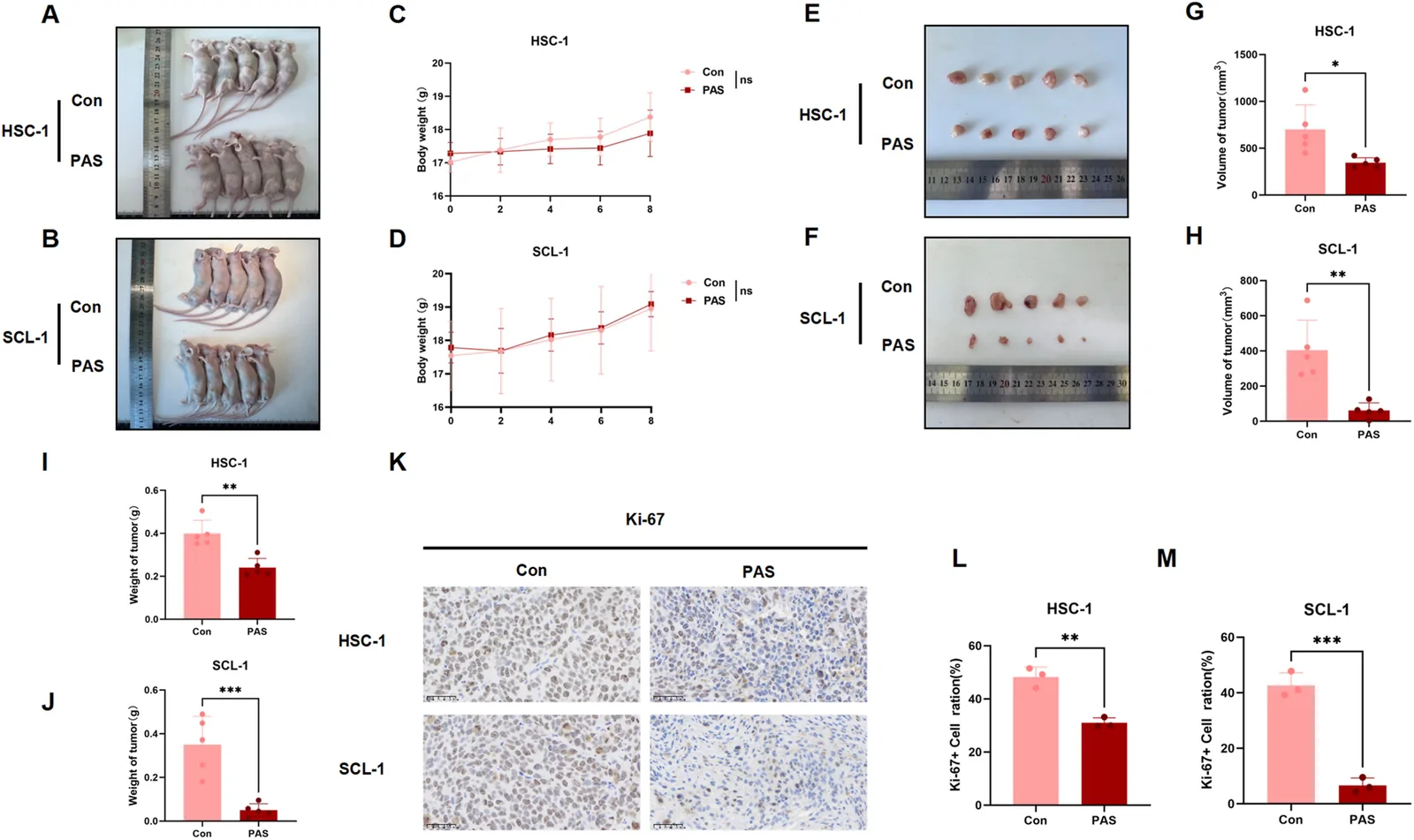
PAS treatment inhibited the growth of transplanted tumors. **(A,B)** Photographs of the mice at the end of the experiment (n = 5); **(C,D)** Changes in the mice’s weight over time; **(E,F)** Photographs of the tumor at the end of the experiment (n = 5); **(G,H)** Quantification of tumor volume; **(I,J)** Quantification of tumor weight; **(K)** Ki-67 staining of the mouse tumor tissues; **(L,M)** Quantification of Ki-67 staining in mouse tumor tissues (Scale bar = 50 μm, n = 3); Mean ± Standard Error of the Mean, n = 3, ∗p < 0.05; ∗∗p < 0.01; ∗∗∗p < 0.001; ∗∗∗∗p < 0.0001.

To evaluate the *in vivo* antitumor efficacy of PAS, HSC-1 and SCL-1 cells were subcutaneously injected into nude mice to establish xenograft tumor models (Figures 2A,B). Throughout the experimental period, mice in the PAS-treated group maintained good general condition, and body weight gain curves showed no significant difference compared with the untreated group (Figures 2C,D), indicating that PAS treatment did not induce appreciable systemic toxicity. Compared with the untreated group, tumor volume growth was significantly suppressed in the PAS-treated group (Figures 2E–H), and terminal tumor weights were also markedly reduced (Figures 2I,J). Immunohistochemical analysis further revealed a significant decrease in Ki-67 positive proliferating cells within tumor tissues of the PAS-treated group (Figures 2K–M), confirming that PAS effectively inhibited tumor cell proliferation *in vivo*.

### 3.3 PAS promoted apoptosis and inhibited migration in xenograft tumors *in vivo*

**FIGURE 3:**
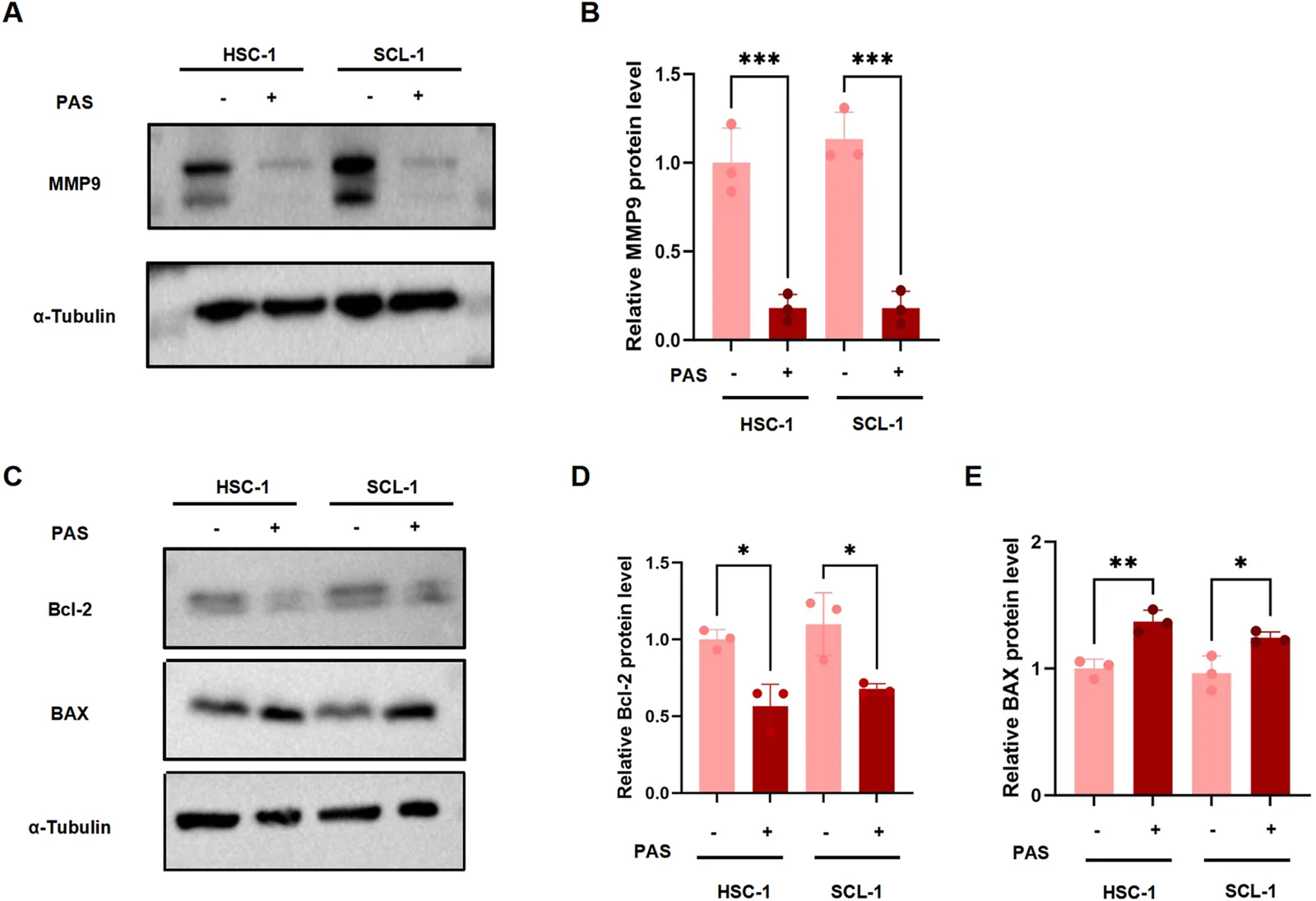
PAS promoted tumor apoptosis and inhibits their migration. **(A)** Western blotting results of MMP9 in mouse tumor tissues; **(B)** Quantification of A protein expression level; **(C)** Western blotting results of Bcl-2 and BAX in mouse tumor tissues; **(D,E)** Quantification of C protein expression level; Mean ± Standard Error of the Mean, n = 3, ∗p < 0.05; ∗∗p < 0.01; ∗∗∗p < 0.001; ∗∗∗∗p < 0.0001.

The results showed that PAS treatment significantly reduced the expression of MMP9 protein (Figures 3A,B). MMP9 is a well-known marker for tumor migration, suggesting that it has the ability to inhibit tumor cell migration. Bax and Bcl-2 are key regulators of apoptosis. Western blotting analysis demonstrated that, compared with the control group, PAS treatment significantly upregulated the pro-apoptotic protein Bax and downregulated the anti-apoptotic protein Bcl-2 in both HSC-1 and SCL-1 xenograft tumors (Figures 3C–E), indicating effective activation of the intrinsic apoptotic pathway. Taken together, these results indicate that PAS promotes apoptosis through modulation of the Bax/Bcl-2 axis and concurrently attenuates MMP9-mediated tumor migration *in vivo*.

### 3.4 PAS inhibited proliferation and promoted apoptosis in cutaneous cSCC cell lines

**FIGURE 4:**
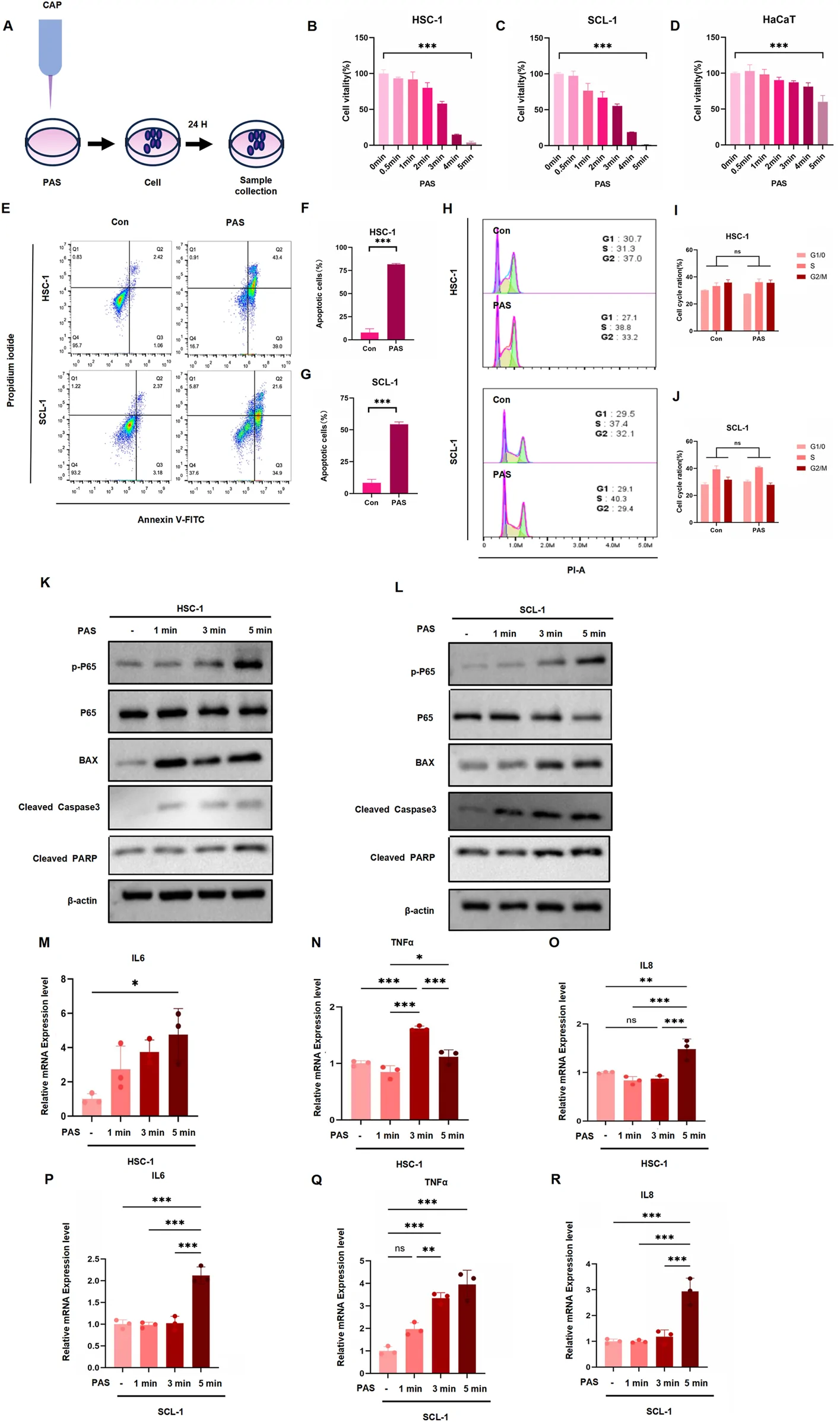
PAS treatment inhibited the survival ability of the tumor and promoted its apoptosis **(A)** Schematic diagram of the *in vitro* experiment of PAS; **(B–D)** Results of CCK8 assay; **(E)** Flow cytometry results of tumor cell apoptosis after PAS treatment; **(F,G)** Quantification of apoptosis level of E cells; **(H)** Flow cytometry results of tumor cell cycle after PAS treatment; **(I,J)** Quantification of H cell cycle **(K,L)** Western blotting results of p-P65, P65, BAX, Cleaved Caspase3 and Cleaved PARP in tumor cells; **(M–R)** mRNA expression of inflammatory factors in tumor cells after PAS treatment; Mean ± Standard Error of the Mean, n = 3, ∗p < 0.05; ∗∗p < 0.01; ∗∗∗p < 0.001; ∗∗∗∗p < 0.0001.

The *in vitro* PAS treatment process is illustrated in Figure 4A. CCK-8 assay results demonstrated that PAS treatment significantly inhibited the survival ability of both HSC-1 and SCL-1 cell lines. Following incubation with PAS generated at varying treatment durations, cell viability decreased in a time-dependent manner with increasing CAP exposure time: compared with the control group, cell viability in both lines decreased by approximately 80% when the PAS treatment duration reached 4 min (Figures 4B,C). In normal keratinocytes (HaCaT cells), the cell viability remains above 50% even after 5 min of PAS treatment (Figure 4D), which further demonstrates the anti-tumor selectivity and biosafety of PAS. To further elucidate the underlying mechanism, apoptosis and cell cycle distribution were assessed by flow cytometry. The results showed that PAS treatment significantly induced apoptosis in both HSC-1 and SCL-1 cells compared with the control (Figures 4E–G). However, no significant alterations in cell cycle distribution were observed in either cell line (Figures 4H–J), indicating that PAS exerted its antitumor effects primarily through apoptosis induction rather than cell cycle arrest.

Meanwhile, Western blot analysis (Figures 4K,L) showed that the expression of pro-apoptotic protein BAX was upregulated, and the levels of Cleaved Caspase3 and Cleaved PARP were elevated after PAS treatment, confirming the activation of the intrinsic apoptotic pathway. Notably, we also observed an increased p-P65/P65 protein ratio, which was further validated by the significantly upregulated mRNA levels of inflammatory cytokines in both cell lines detected via RT-qPCR (Figures 4M–R), indicating the activation of the NF-κB signaling pathway. The detailed quantitative results of Western blotting are presented in the newly uploaded Supplementary Data Sheet 1. 

### 3.5 PAS induced oxidative stress in HSC-1 and SCL-1 cells

**FIGURE 5:**
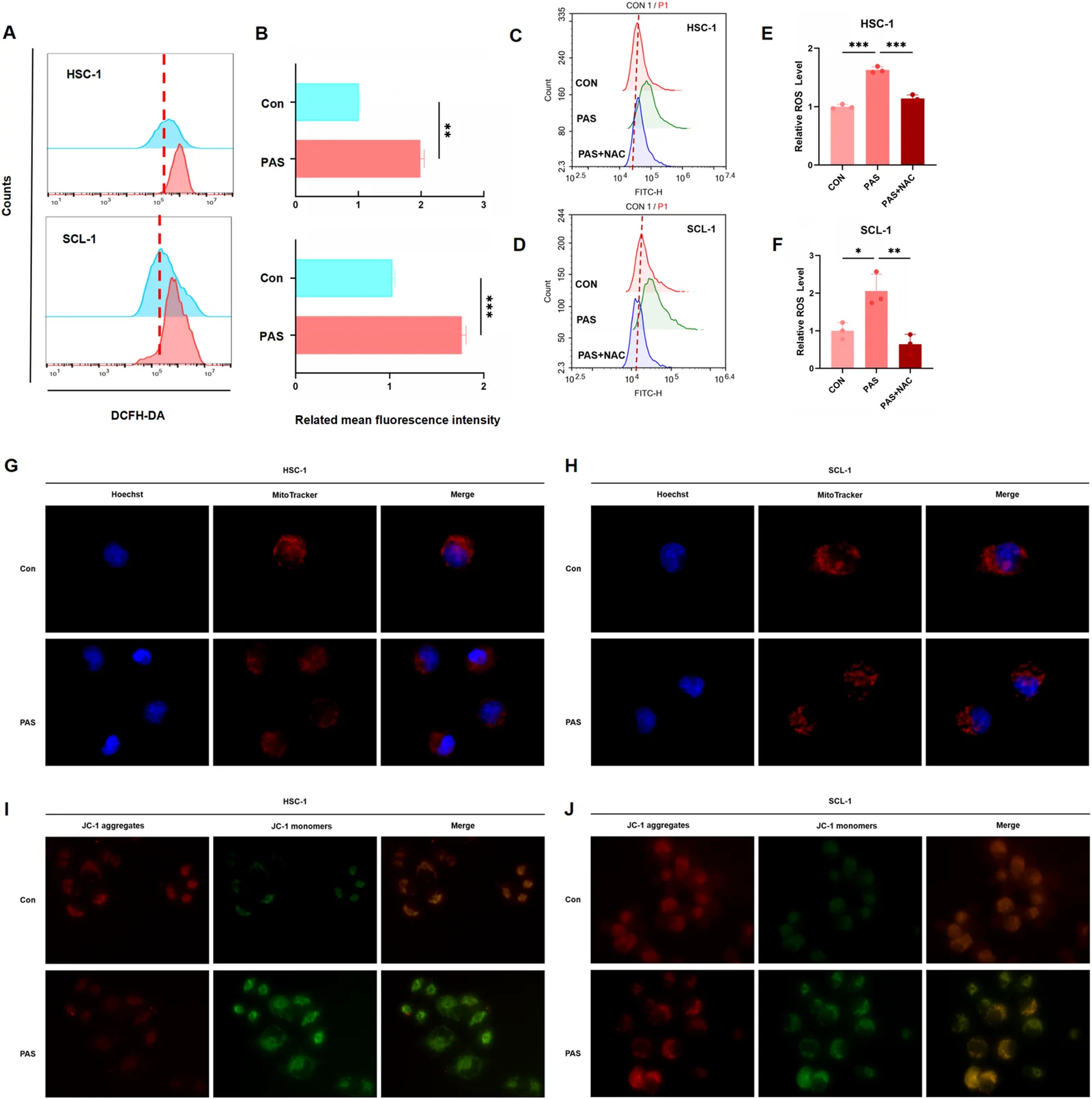
Oxidative stress damage to tumor cells caused by PAS treatment. **(A)** Flow cytometry analysis of intracellular ROS levels in tumor cells after PAS treatment; **(B)** Results of quantifying ROS levels through fluorescence geometric mean; **(C,D)** Flow cytometry analysis of intracellular ROS levels in tumor cells after NAC treatment; **(E,F)** Results of quantifying ROS levels through fluorescence geometric mean; **(G,H)** MitoTracker red staining results of tumor cells (Scale bar = 20 μm); **(I,J)** JC-1 mitochondrial membrane potential images (Scale bar = 50 μm); Mean ± Standard Error of the Mean, n = 3, ∗p < 0.05; ∗∗p < 0.01; ∗∗∗p < 0.001; ∗∗∗∗p < 0.0001.

Cold atmospheric plasma treatment generates substantial quantities of highly reactive oxygen/nitrogen species (ROS/RNS), thereby inducing oxidative stress in cells and triggering mitochondria-dependent apoptosis through ROS accumulation. To delineate the upstream mechanisms of PAS-induced apoptosis, intracellular ROS levels were first assessed. Flow cytometric analysis revealed that PAS treatment significantly elevated ROS levels in both HSC-1 and SCL-1 cell lines compared with the control (Figures 5A,B). To clarify the core mediating role of reactive oxygen species (ROS) in the tumor-killing effect induced by PAS, this study performed a rescue verification experiment using the classic ROS scavenger N-acetylcysteine (NAC). Flow cytometry results showed that compared with the blank control group, PAS treatment significantly upregulated the intracellular ROS level in HSC-1 and SCL-1 cells; while after adding NAC to the PAS treatment system, the abnormally elevated ROS levels in the two tumor cell lines were effectively reversed (Figures 5C–F). Meanwhile, MitoTracker red staining and JC-1 staining verified that PAS treatment led to decreased mitochondrial membrane potential and abnormal mitochondrial morphology (Figures 5G–J), indicating that mitochondrial dysfunction is a critical link in PAS-induced apoptosis.

### 3.6 PAS suppressed migration and invasion of cSCC cells

**FIGURE 6:**
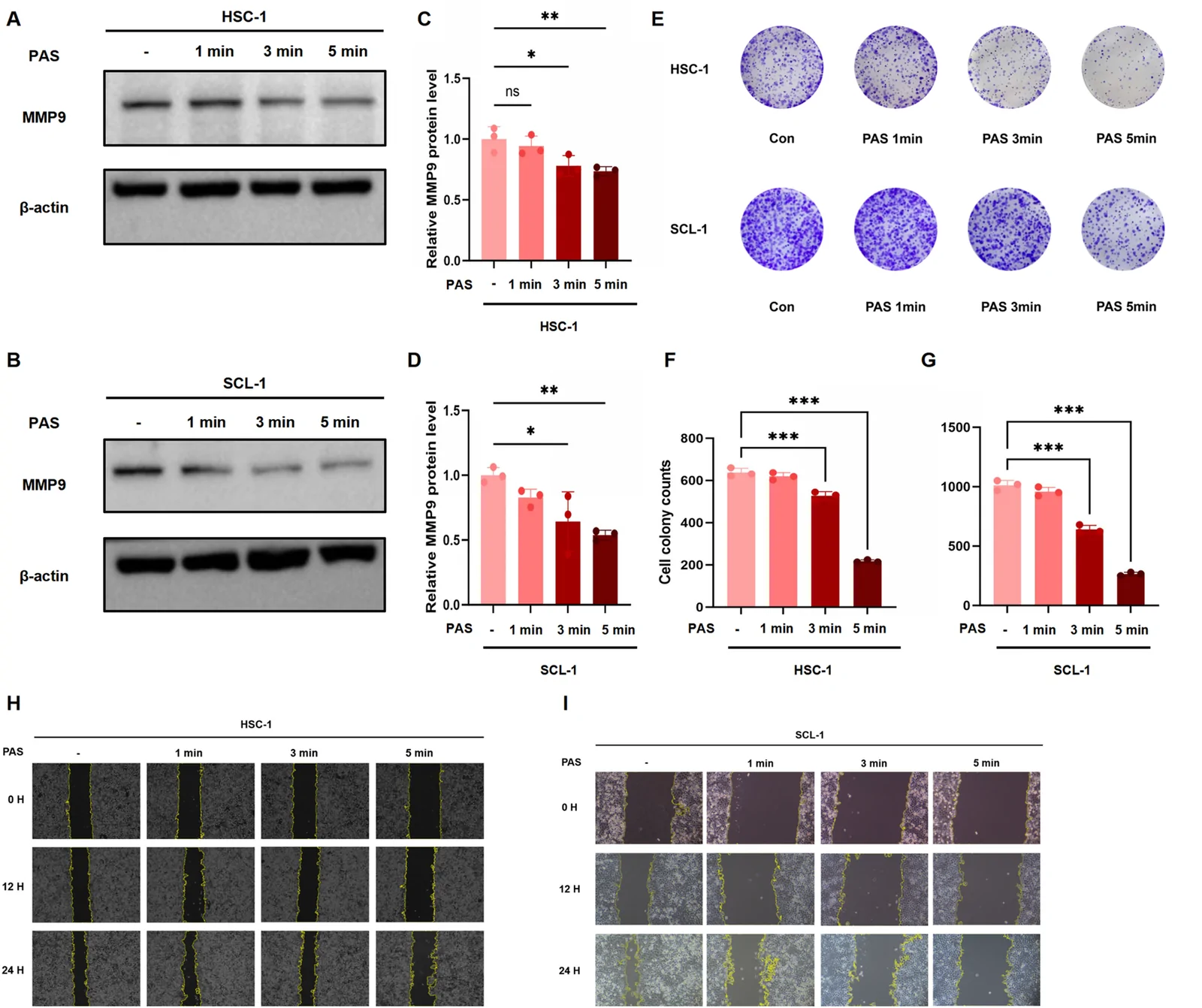
PAS treatment inhibited the migration and invasion ability of cells. **(A,B)** Western blotting results of MMP9 in tumor cells; **(C,D)** Quantification of protein expression levels; **(E)** Clonogenic formation results of tumor cells after PAS treatment; **(F,G)** Quantification of the number of cell clones; **(H,I)** Scratch assay results of tumor cells after PAS treatment; Mean ± Standard Error of the Mean, n = 3, ∗p < 0.05; ∗∗p < 0.01; ∗∗∗p < 0.001; ∗∗∗∗p < 0.0001.

Matrix metalloproteinase-9 (MMP9), a key effector molecule involved in tumor migration and invasion, was significantly downregulated at the protein level following PAS treatment, as confirmed by Western blotting (Figures 6A–D). We subsequently examined the effects of PAS treatment on the migratory and invasive capacities of HSC-1 and SCL-1 cells. Colony formation (Figures 6E–G) and wound healing assays (Figures 6H,I) revealed that both clonogenic capacity and migration rate decreased in a time-dependent manner with increasing CAP treatment duration. Collectively, these findings demonstrated that PAS effectively suppressed the migration and invasion of cutaneous SCC cells through downregulation of MMP9 expression.

### 3.7 Safety assessment of PAS

**FIGURE 7:**
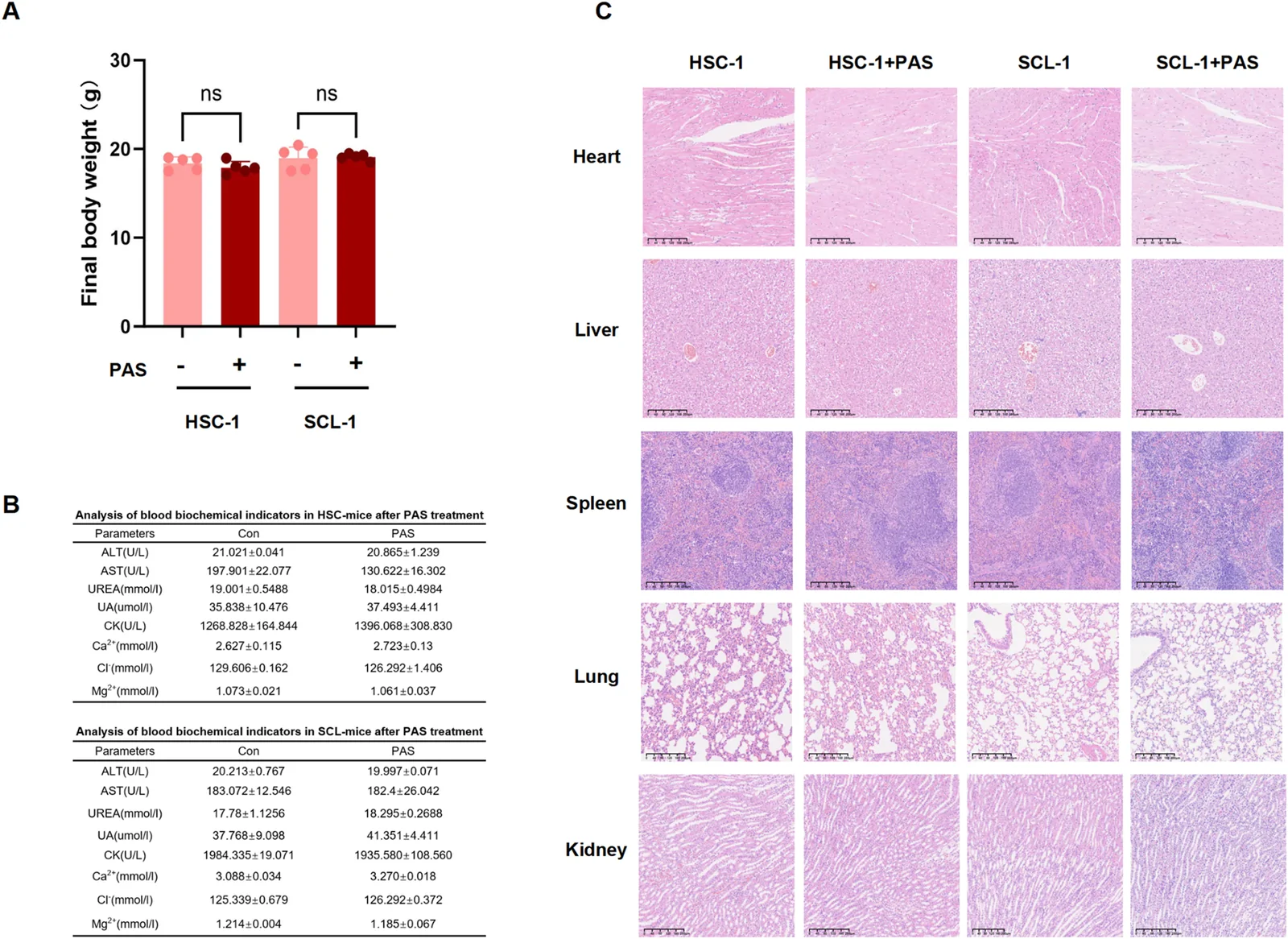
Safety assessment of PAS. **(A)** Mouse body weight at the end of the experiment (n = 5); **(B)** Mouse blood biochemical indicators; **(C)** H&E staining of important organs of mice (Scale bar = 200 μm); Mean ± Standard Error of the Mean, ∗p < 0.05; ∗∗p < 0.01; ∗∗∗p < 0.001; ∗∗∗∗p < 0.0001.

To evaluate the *in vivo* safety of PAS, general condition was monitored continuously throughout the experiment, and serum biochemical and histopathological analyses were performed at the experimental endpoint. Mice in the PAS-treated group maintained good general condition with stable body weight gain over the course of the study, and no significant difference in final body weight was observed between groups (Figure 7A). Serum biochemical analysis revealed no significant differences between the PAS-treated and control groups in aspartate aminotransferase (AST), alanine aminotransferase (ALT), Serum Urea (UREA), uric acid (UA), creatine kinase (CK), or major inorganic ion levels (Figure 7B), suggesting that PAS administration did not cause notable hepatorenal injury or cardiotoxicity. Gross anatomical examination also revealed no abnormal organ adhesions in either group. Moreover, H&E staining analysis demonstrated no evident pathological changes in the heart, liver, spleen, lung, and kidney of PAS-treated mice (Figure 7C), consistent with the control group. Collectively, these results confirmed the favorable biosafety profile of PAS under the experimental conditions employed.

## 4 Discussion

Cold atmospheric plasma (CAP) has been applied to a wide range of diseases and has gained extensive attention in oncology in recent years, encompassing lung cancer, gastric cancer, colorectal cancer, and cutaneous malignancies (Liu et al., 2024; Zhang et al., 2024; Qi et al., 2025; Chen et al., 2024; Ito et al., 2025). Compared with direct CAP treatment, plasma-activated solution (PAS) offers superior safety, enhanced portability, greater patient acceptability, and more convenient application to body cavities or tumor regions that are not directly accessible to plasma (Qi et al., 2025; Min et al., 2026).

In the present study, we established the therapeutic potential of PAS for cutaneous squamous cell carcinoma (cSCC). Our *in vivo* experiments demonstrated significant antitumor efficacy of PAS in a nude mouse xenograft model, including suppression of tumor growth, reduction of Ki-67-positive proliferating cells, and absence of body weight loss or organ damage.

Regarding the antitumor mechanism, aberrant proliferation and apoptosis resistance in cSCC cells constitute core drivers of tumor progression (Jia et al., 2023; Welponer et al., 2024). Our results demonstrated that PAS significantly inhibited the proliferation of both HSC-1 and SCL-1 cell lines in a time-dependent manner, with cell viability decreasing by approximately 80% after 4 min of CAP treatment. The experimental data demonstrated that normal HaCaT cells exhibited significantly lower sensitivity to PAS treatment compared to the tested malignant tumor cell lines under equivalent experimental conditions. This differential cytotoxicity profile provides direct *in vitro* evidence supporting that PAS possesses a certain degree of selective anti-tumor potency, along with favorable safety performance toward normal human skin-derived cells. Flow cytometric analysis revealed that PAS exerted its antiproliferative effects primarily through apoptosis induction rather than cell cycle arrest. Western blotting analysis further confirmed that PAS significantly upregulated the pro-apoptotic protein Bax and downregulated the anti-apoptotic protein Bcl-2, and the levels of Cleaved Caspase3 and Cleaved PARP were elevated after PAS treatment, indicating that PAS activates the intrinsic apoptotic pathway via modulation of the Bax/Bcl-2 axis.

At physiological concentrations, ROS play multifaceted regulatory roles in cell signaling, whereas excessive ROS induces oxidative stress damage and triggers cell death (Zhou et al., 2026). Within ROS-mediated signaling cascades, mitochondria serve as critical effector targets (Foo et al., 2022; Rivadeneira et al., 2025; Wen et al., 2025). In this study, PAS treatment significantly elevated intracellular ROS levels in both HSC-1 and SCL-1 cells, and this effect could be alleviated by the classical ROS scavenger NAC. MitoTracker red staining and JC-1 staining further confirmed that PAS induced a decrease in mitochondrial membrane potential and abnormal mitochondrial morphology, indicating that mitochondrial dysfunction is a pivotal upstream event in PAS-induced apoptosis. ROS accumulation can execute apoptosis through the Bax/Bcl-2 axis (Qi et al., 2023).

In addition to direct apoptosis induction, activation of the NF-κB signaling pathway was also observed in this study. PAS treatment increased p-P65/P65 protein expression, and RT-qPCR detected a significant upregulation of inflammatory cytokine mRNA levels. As a redox-sensitive transcription factor, NF-κB pathway can be activated by oxidants and metabolic stresses (Averill-Bates, 2024). In this study, the high-intensity ROS burst induced by PAS may disrupt the conventional pro-survival regulatory mode of NF-κB: the excessively accumulated oxidative stress signal synergizes with the activated NF-κB pathway to jointly amplify the transcriptional cascade effect of downstream apoptosis-related signals, ultimately driving tumor cells into apoptosis. This potential synergistic regulatory mechanism still requires further validation in subsequent studies.

Tumor migration and invasion are the primary causes of cSCC recurrence and metastasis. Matrix metalloproteinase-9 (MMP9), a key effector molecule involved in extracellular matrix degradation and tumor invasion, is closely associated with poor prognosis in multiple cancers (Owecki and Nijakowski, 2025; Jiang and Li, 2021; Abbasi et al., 2026). In this study, PAS treatment significantly downregulated MMP9 protein expression, and both colony formation and wound healing assays confirmed that PAS suppressed cell migration in a time-dependent manner. These findings indicate that PAS not only inhibits primary tumor growth but may also restrict the invasive and metastatic potential of tumor cells through MMP9 downregulation, which carries significant clinical implications for reducing cSCC recurrence risk.

Regarding safety, a systematic *in vivo* safety assessment was conducted. Mice in the PAS-treated group exhibited stable body weight gain, with no significant difference in terminal body weight compared with the control group. Serum biochemical analysis revealed that AST, ALT, UREA, UA, CK, and major inorganic ion levels remained within normal ranges, suggesting no appreciable hepatorenal injury or cardiotoxicity. H&E staining demonstrated no pathological changes in the heart, liver, spleen, lung, or kidney. These results are consistent with the favorable safety profile of PAS previously reported in inflammatory skin disease models, further supporting the feasibility of PAS as a local antitumor therapeutic strategy (Yin et al., 2024).

Notably, long-lived reactive species in PAS, such as H_2_O_2_ and nitrite, are capable of penetrating biological barriers and play critical roles in both *in vitro* and *in vivo* applications (Qi et al., 2025). H_2_O_2_ has been identified as the predominant ROS species mediating bioactive redox regulation (Kračun et al., 2025). This underscores the potential of PAS for selective tumor cell killing: tumor cells typically exhibit a higher basal oxidative stress level and are consequently more vulnerable to additional ROS insults, whereas normal cells, equipped with more robust antioxidant defense systems, can tolerate a certain concentration of ROS exposure (Kawaguchi et al., 2025). By contrast, short-lived species such as hydroxyl radicals and singlet oxygen, despite their extremely high reactivity, have limited therapeutic efficacy due to their transient nature. Therefore, the antitumor effects of PAS are primarily attributable to long-lived reactive species such as H_2_O_2_ (Yin et al., 2024).

This study has several limitations that warrant further investigation. First, only two cSCC cell lines (HSC-1 and SCL-1) and a subcutaneous xenograft model in nude mice were employed; the therapeutic efficacy of PAS against other cSCC subtypes or orthotopic tumor models remains to be determined. Second, the downstream signaling cascades triggered by PAS-induced ROS accumulation remain incompletely characterized. More critically, the downstream signaling cascades triggered by PAS-induced ROS accumulation remain incompletely characterized. Although oxidative stress has been established as a key effector, the precise molecular targets have not been systematically dissected. Identifying the direct binding targets of PAS-derived active compounds through thermal proteome profiling or CETSA is essential for distinguishing on-target from off-target effects and for elucidating structure-activity relationships.

From a translational perspective for application, several critical barriers remain to be addressed. The local stability of PAS under physiological conditions, its optimal storage conditions, and batch-to-batch reproducibility in manufacturing require rigorous characterization to ensure consistent therapeutic efficacy. Determining a standardized dosing metric for its reactive species output, rather than a simple molar concentration, is crucial for clinical translation. Furthermore, key pharmacodynamic parameters for local therapy—including its tissue penetration depth, intratumoral distribution following injection, the appropriate injection volume, and compatibility with clinical delivery devices—are currently unknown and must be defined. Finally, a thorough evaluation of long-term local toxicity and the potential for adverse reactions in surrounding normal tissue is imperative before clinical trial design.

Future work should therefore prioritize establishing Good Manufacturing Practice (GMP)-compliant production, standardizing the reactive dose, evaluating local pharmacokinetics/pharmacodynamics in immunocompetent models, and conducting GLP-complanted local toxicity studies to bridge the gap between the observed preclinical efficacy and clinical applicability for cSCC treatment.

## 5 Conclusion

In summary, this study demonstrates that PAS exerts antitumor effects through a core “ROS–mitochondrial damage–Bax/Bcl-2 apoptosis axis' and suppresses tumor migration and invasion via MMP9 downregulation, combining therapeutic efficacy with a favorable safety profile. These findings provide an experimental foundation for the further development of PAS as an adjuvant therapeutic strategy for cSCC. Future studies should validate its broad-spectrum applicability across additional cSCC models and conduct pharmacokinetic and preclinical translational investigations.

## Data Availability

The original contributions presented in the study are included in the article/Supplementary Material, further inquiries can be directed to the corresponding authors.
