# Supplementary material for: Inhibitory effects of cold atmospheric plasma-activated solution on cutaneous squamous cell carcinoma
Source: Front Pharmacol. 2026 Sep 11;17:1921696. doi: 10.3389/fphar.2026.1921696 (PMC13612911; doi:10.3389/fphar.2026.1921696)
Supplement: Supplementary file 1 [file DataSheet1.pdf]

Fig.4K-L

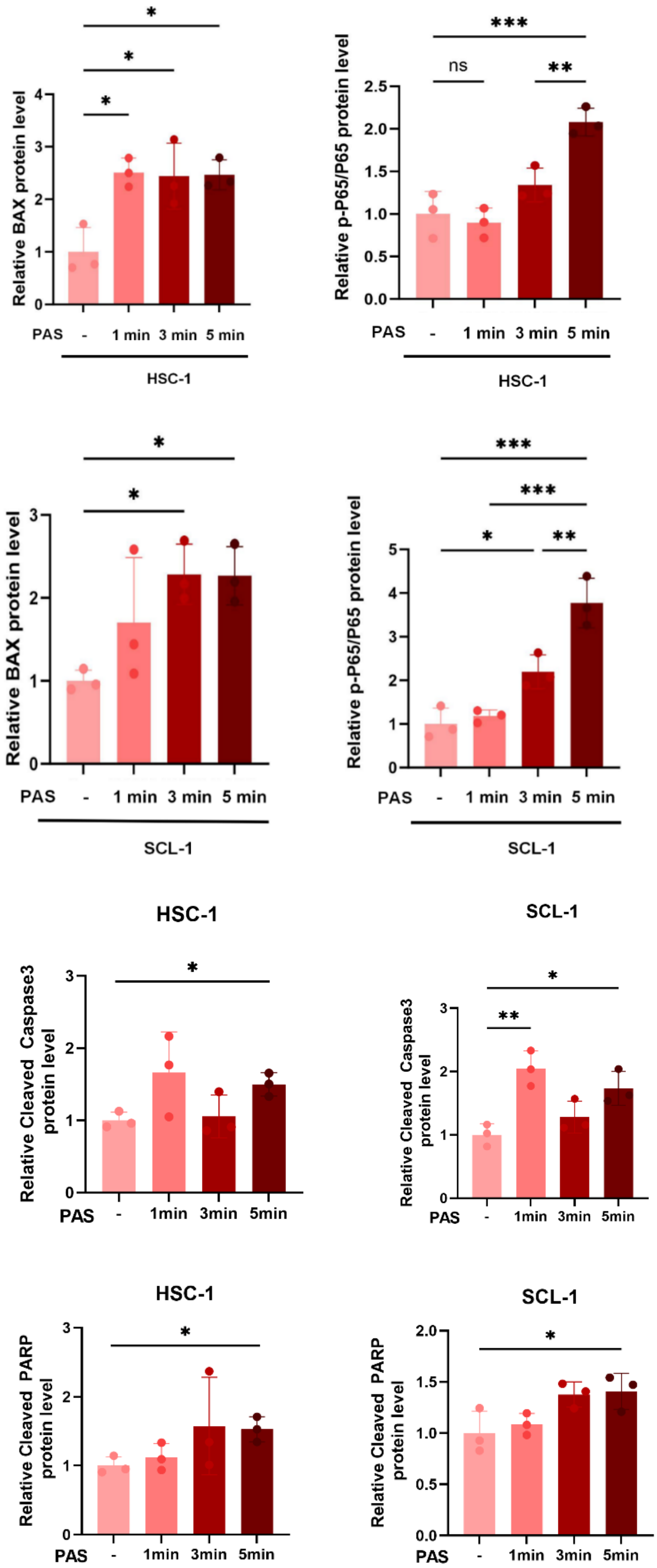
Relative BAX protein level
PAS - 1 min 3 min 5 min
HSC-1
Relative p-P65/P65 protein level
ns
PAS - 1 min 3 min 5 min
HSC-1
Relative BAX protein level
PAS - 1 min 3 min 5 min
SCL-1
Relative p-P65/P65 protein level
PAS - 1 min 3 min 5 min
SCL-1
HSC-1
Relative Cleaved Caspase3 protein level
PAS - 1min 3min 5min
SCL-1
Relative Cleaved Caspase3 protein level
PAS - 1min 3min 5min
HSC-1
Relative Cleaved PARP protein level
PAS - 1min 3min 5min
SCL-1
Relative Cleaved PARP protein level
PAS - 1min 3min 5min
